# Preparation and Evaluation of a Horse Antiserum against the Venom of Sea Snake *Hydrophis curtus* from Hainan, China

**DOI:** 10.3390/toxins14040253

**Published:** 2022-04-01

**Authors:** Bo Wang, Guoyan Liu, Min Luo, Xin Zhang, Qianqian Wang, Shuaijun Zou, Fuhai Zhang, Xia Jin, Liming Zhang

**Affiliations:** 1 Department of Marine Biomedicine and Polar Medicine, Naval Characteristic Medical Center, Naval Medical University, Shanghai 200433, China; m18817365409@163.com (B.W.); lgy_laurie@aliyun.com (G.L.); abc_w@163.com (Q.W.); smmuzsj@163.com (S.Z.); zhangfh1105@163.com (F.Z.); 2 Shanghai Serum Bio-Technology Co., Ltd., Shanghai 201707, China; luomin@serum-china.com (M.L.); zhangxin@serum-china.com.cn (X.Z.)

**Keywords:** *Hydrophis curtus*, sea snake venom, antivenom, first aid

## Abstract

Sea snake venom is extremely toxic, and it can induce severe respiratory failure and cause high mortality. The most effective first aid treatment for sea snake bites is to inject antivenom as soon as possible. However, in China, there are only four types of terrestrial snake antivenoms, none of which are effective in the treatment of sea snake bites. In order to develop an antivenom for the dominant species of sea snakes in Chinese seas, *Hydrophis curtus* venom (HcuV) was chosen as the antigen to immunize horses. From immune plasma, a high-titer *Hydrophis curtus* antivenom (HcuAV) was prepared. In vitro assessment showed that HcuAV had a cross-neutralizing capacity against HcuV and *Hydrophis cyanocinctus* venom (HcyV). In vivo assessment indicated that HcuAV injection could significantly improve the survival rates of the HcuV and HcyV envenomated mice (0% to 100% and 87.5%, respectively) when it was injected at a sufficient amount within the shortest possible time. In addition, HcuAV could also effectively alleviate multiple organ injuries caused by HcuV. These results provide experimental support for the future clinical application of HcuAV.

## 1. Introduction

Snakebites have always been a very important public medical problem in tropical and subtropical regions of the world [1,2,3]. An estimated 1.8–2.7 million snakebite cases occur worldwide each year, causing 80,000–130,000 deaths [4]. Despite the relatively high mortality, it was only in recent years that snakebite envenomation was officially named by the World Health Organization as a tropical and subtropical disease that cannot be ignored, and this attracted widespread attention [3]. The clinical symptoms of snakebite envenomation vary according to the types of snake venom [5], which can be divided into three categories: (I) venom with toxicity to the circulation system [6,7], including the induction of cardiotoxicity and blood-circulation disorders; (II) venom with neurotoxicity [8,9], which can cause muscle paralysis by blocking nerve synaptic transmission; (III) venom with mixed toxicity [10,11], which exhibits both blood-circulation toxicity and neurotoxicity.

As one of the most dangerous marine creatures, sea snakes are all highly venomous [12]. However, the studies on sea snakes are lagging behind their terrestrial counterparts, and the existing reports mostly focus on a few species of sea snakes, such as *Hydrophis curtus*, *Enhydrina schistosa*, *Hydrophis platurus* and *Laticauda colubrina*, etc. [12,13,14,15]. These studies demonstrated that sea snake venoms are mixtures of various enzymes and polypeptides. The enzymes include phospholipase A2, metalloprotease, serine protease, L-amino acid oxidase, and hyaluronidase, which may play the role of digestion and promote toxin diffusion [16], and the polypeptides are mainly α-neurotoxins of the three-finger toxin (3FTx) family, which can be divided into short-chain neurotoxins (SNTX) and long-chain neurotoxins (LNTX) [17,18]. The α-neurotoxins can bind to the acetylcholine receptors of the postsynaptic membrane and block neuromuscular transmission, so sea snake venoms exhibit typical neurotoxicity [13,16,19].

Sea snakes are generally docile and do not actively attack humans. The incidence of sea snake bites is lower than that of terrestrial snake bites. However, sea snake bites usually occur during sea operations or in remote fishing villages; therefore, the statistical data of sea snake bites might be lower than the actual incidence. Most sea snake envenomated patients exhibit mild local symptoms and insignificant pain, and thus they are easily ignored [20,21,22]. Systemic symptoms usually appear 0.5 h to 2 h after the bite. Early symptoms mainly include muscle dissolution and paralysis, and some patients may develop acute renal failure. Due to the toxic effect of neurotoxins, patients will suffer from rapidly progressive muscle paralysis symptoms, leading to irreversible respiratory failure [23]. 

As a relatively common public medical problem, there are a few standardized treatments for snakebites [24], among them the most effective treatment is to inject specific antivenom as soon as possible [25,26,27]. If specific antivenom can be obtained in time, most of the deaths caused by snakebites can be avoided. Currently, the only sea snake antivenom on the market worldwide is Antivenin *Enhydrina schistosa*. This antivenom has been confirmed to have a good neutralization effect on the venoms of some species of terrestrial snakes and sea snakes [15,28,29], but it remains difficult to obtain Antivenin *Enhydrina schistosa* in some regions. Compared with terrestrial snake antivenoms, the types of sea snake antivenoms are still very scarce in the world [30]. In China, there are just four types of terrestrial snake antivenoms on sale, namely *Naja atra* antivenom (NaAV), *Agkistrodon halys* antivenom (AhAV), *Deinagkistrodon acutus* antivenom (DaAV), and *Bungarus multicinctus* antivenom (BmAV). Most of sea snake bite cases in China have to be treated with the above terrestrial snake antivenoms, which can lead to unsatisfactory effects and some adverse reactions. The lack of specific sea snake antivenom poses a great danger to fishermen and underwater workers. Therefore, it is urgent to develop an antivenom specifically against the dominant species of sea snakes in Chinese seas.

*Hydrophis curtus* and *Hydrophis* *cyanocinctus* are the two dominant species of sea snakes in Chinese seas [13], and a previous study by our group has indicated that the venom compositions of HcuV were similar to HcyV [23]. In the present study, we chose HcuV as the antigen to prepare a horse antivenom and evaluated the cross-neutralization of HcuAV against sea snake venoms in vivo and in vitro. The results of this study provide the scientific basis for the future clinical application of HcuAV.

## 2. Results

### 2.1. Kinetic Changes of Immune Titer in Plasma after Immunization

To determine the optimal schedule of immunization, plasma was collected from four horses after each immunization and tested for activities against venom antigens. The immune titer of the collected plasma increased progressively from 10 to 50 d, and exceeded an absorbance of 1.5 at 450 nm, an arbitrary set titer at which plasma could be collected for the preparation of antivenom-products (Table 1).

### 2.2. Cytotoxicity Assay of the Immune Plasma

**Figure 1:**
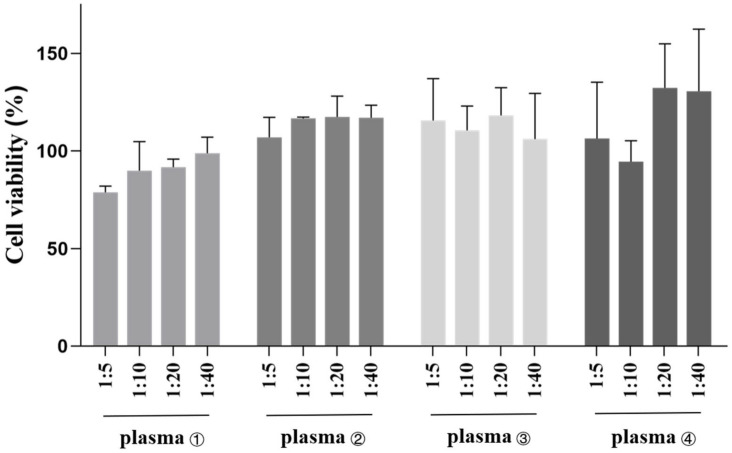
Effects of the collected horse plasma on the viability of Vero cells at different dilutions. Briefly, Vero cells were treated with different dilutions of the immune plasma (at 1:5, 1:10, 1:20, 1:40) for 12 h. Then, 10 μL of CCK-8 reagents was added to each well for 2 h at 37 °C. Absorbance at 450 nm was measured using a microplate reader. All treatments were performed in triplicate individually, and the data are presented as means ± SD (*n* = 3).

To evaluate the biosafety of the immune plasma, Vero cells (African green monkey kidney cells) were used to perform a cytotoxicity assay. As shown in Figure 1, after 12 h of the treatment with the horse plasma No. 1–4, Vero cells maintained a viability of above 80%, and certain groups even showed increased cell proliferation, demonstrating that the collected horse plasma had no obvious cytotoxicity.

### 2.3. Purification of HcuAV and Quantitative Analysis

Antivenom immunoglobulins were then prepared from the plasma of fully immunized horses. Immune plasma contains intact IgG and some high molecular protein impurities, and thus, it is easy to cause allergic reactions. In addition, the Fc fragment may also promote the formation of immune complexes, which is one of the main causes of allergic reactions. Therefore, enzymatic digestion and purification of the immune plasma are important for antivenom production to remove the intact IgG and digestion fragments. 

**Figure 2:**
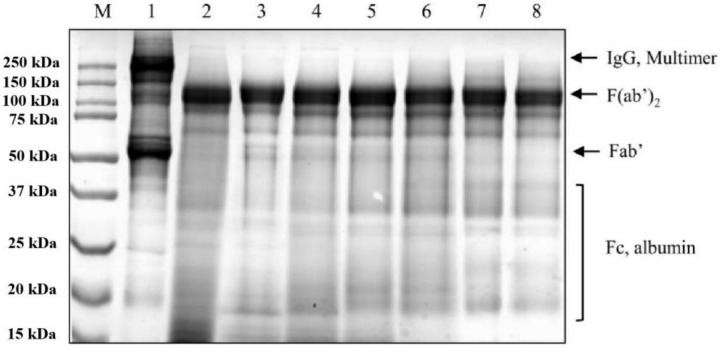
Tris-SDS-PAGE analysis of each sample during antibody purification. Lane 1–8: immune plasma, enzyme digestion solution, supernatant I after primary precipitation, precipitation resuspension after secondary precipitation, supernatant II after alum adsorption, ultrafiltrate, stock solution, and end product (HcuAV).

Briefly, the enzymatic digestion and purification process included the following steps: pepsin digestion, primary precipitation with ammonium sulfate, secondary precipitation, alum adsorption, ultrafiltration of the supernatant, and DEAE column chromatography. The antivenom stock solution was finally obtained by filtering and sterilizing through a 0.22 μm filter. Each sample of HcuAV during purification was loaded on SDS-PAGE gel for electrophoretic analysis. As shown in Figure 2, as the purification progressed, the purity of F(ab’)_2_ (molecular weight approximately 110 kDa) gradually improved, and the contaminants (Fab’, Fc, multimer, and albumin fragments) were gradually removed.

To make a quantitative analysis, we further performed HPLC analysis on the samples during purification. As shown in Table 2, the proportion of F(ab’)_2_ in the sample reached 91.6% after purification, a relatively high-purity for an HcuAV product.

### 2.4. Binding Ability of HcuAV towards Various Snake Venoms

To examine the sensitivity and specificity of HcuAV, indirect ELISA and diffusion assays were performed. For the indirect ELISA assay, venoms were coated in 96-well plates with a concentration of 10 μg/mL, and antivenoms were diluted at 1: 1000 as the antibody. For the diffusion assay, undiluted HcuAV and a concentration of 1 mg/mL of various snake venoms was added to each well with a volume of 10 μL and then incubated at 37 °C for 12 h.

**Figure 3:**
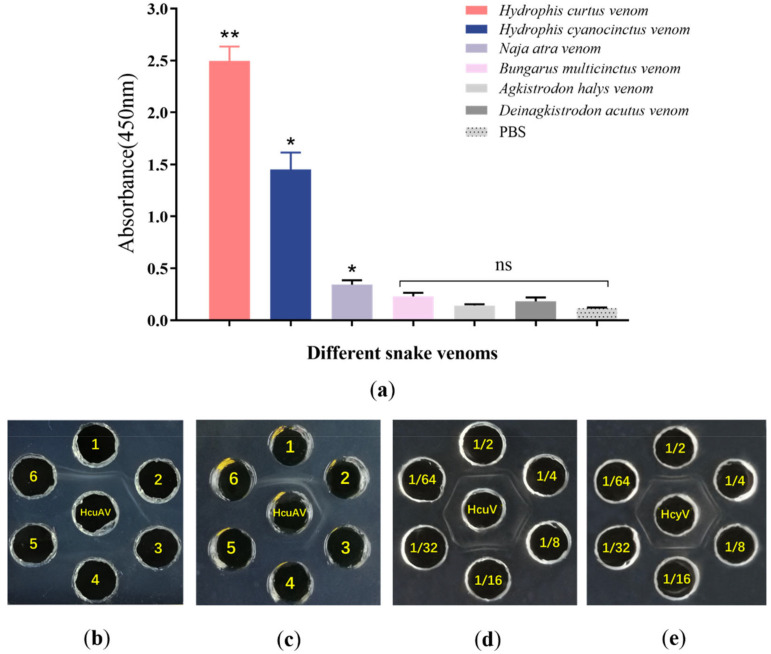
Binding ability of HcuAV towards various snake venoms. In the indirect ELISA assay, venoms were coated in 96-well plates with a concentration of 10 μg/mL, and antivenoms were diluted at 1: 1000 as the antibody. After a series of procedures including coating, antibody binding, washing, and chromogenic reaction, absorbance at 450 nm was measured. For the diffusion assay, undiluted HcuAV and various snake venoms (1 mg/mL) were added to each well with a volume of 10 μL and then incubated at 37 °C for 12 h. (**a**) The binding ability of HcuAV towards various snake venoms by ELISA. Absorbance values were obtained by indirect ELISA and expressed as mean ± SD from three individual experiments (* *p* < 0.05, ** *p* < 0.01, ns: no significance vs. PBS group). (**b**) Immunological assessment of HcuAV against HcuV and HcyV by a Double Immunodiffusion (DID) assay. In the peripheral 1–6 wells: HcuV, HcyV, 2% BSA, PBS, blank, blank. (**c**) Immunological assessment of HcuAV against HcuV, HcyV, and four types of terrestrial snake venoms by a DID assay. In the peripheral 1–6 wells: HcuV, HcyV, *Naja atra* venom (NaV), *Bungarus multicinctus* venom (BmV), *Agkistrodon halys* venom (AhV), and *Deinagkistrodon acutus* venom (DaV). (**d**,**e**) Immunological assessments of different dilutions (1:2, 1:4, 1:8, 1:16, 1:32, 1:64) of HcuAV against HcuV and HcyV by a DID assay.

As shown in Figure 3a, the binding ability of HcuAV towards HcuV and HcyV was significantly stronger than that towards the terrestrial snake venoms. As shown in Figure 3b,c, there were clearer precipitation lines between HcuAV and the two sea snake venoms compared with the four types of terrestrial snake venoms, PBS, and 2% BSA. These results demonstrated that HcuAV exhibited a specific cross-neutralization ability towards HcuV and HcyV, while without obvious binding ability with the terrestrial snake venoms.

As shown in Figure 3d,e, when the dilution of HcuAV reached 1:64, there were still clear precipitation lines in the reactions with HcuV and HcyV, indicating that both the immune titers of HcuAV towards HcuV and HcyV exceeded 1:64.

**Figure 4:**
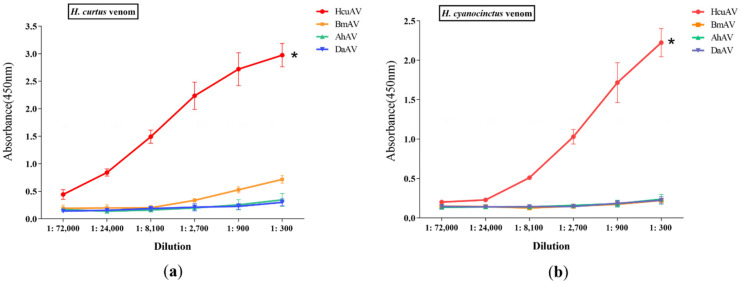
The binding ability of different dilutions of antivenoms towards HcuV (**a**) and HcyV (**b**) by indirect ELISA assay. Briefly, HcuV and HcyV were coated in 96-well plates with a concentration of 10 μg/mL, and then various antivenoms were diluted at 1:72,000 to 1:300 as the antibody. After a series of procedures, including coating, antibody binding, washing, and chromogenic reaction, absorbance at 450 nm was measured using a microplate reader. Absorbance values were expressed as mean ± SD from three individual experiments. (* *p* < 0.05 vs. three terrestrial antivenoms).

To confirm the specificity of HcuAV against sea snake venoms, an ELISA assay was performed using different antivenoms (HcuAV, *Bungarus multicinctus* (elapid family) antivenom, *Agkistrodon halys* (viper family) antivenom, and *Deinagkistrodon acutus* (viper family) antivenom) and two antigens (HcuV and HcyV). Briefly, HcuV and HcyV were coated in 96-well plates with a concentration of 10 μg/mL, and then HcuAV and three types of terrestrial snake antivenoms were diluted at 1:72,000 to 1:300 as the antibody, respectively. After a series of procedures, including coating, antibody binding, washing, and chromogenic reaction, absorbance at 450 nm was measured using a microplate reader. As shown in Figure 4a,b, the absorbance at 450 nm significantly increased as the concentration of HcuAV increased from 1:72,000 to 1:300, while no significant changes of absorbance were observed in the three types of terrestrial snake antivenoms, suggesting that HcuAV had obvious advantages in neutralizing sea snake venoms.

**Figure 5:**
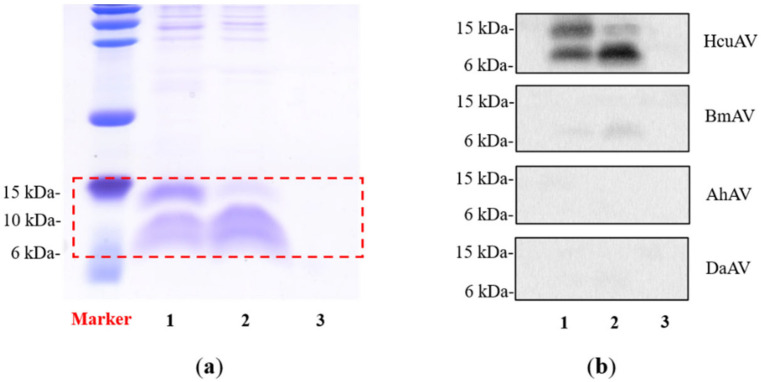
HcuV and HcyV samples were loaded on SDS-PAGE gel and then incubated with HcuAV and three types of terrestrial snake antivenoms at a dilution of 1:1000, respectively, for Western blot analysis. After incubation with the HRP-label antibody, the results were exposed using a chemiluminescent detection system. (**a**) Tris-SDS-PAGE of HcuV and HcyV. Lane 1–3: HcuV, HcyV, blank. (**b**) Western blot analysis of HcuV and HcyV treated with different types of antivenoms. Lane 1–3: HcuV, HcyV, blank.

To explore the main components, which can be recognized by HcuAV in sea snake venoms, a Western blot assay was performed. Briefly, equal amounts of HcuV and HcyV samples were loaded on SDS-PAGE gel and then incubated with HcuAV and three types of terrestrial snake antivenoms at a dilution of 1:1000, respectively, after the transmembrane process. As shown in Figure 5a,b, the two main components of HcuV and HcyV, presumed to be 3FTx (6–10 kDa) and phospholipase A2 (13–16 kDa), could be effectively recognized by HcuAV, but not recognized by the three types of terrestrial snake antivenoms.

### 2.5. Neutralization Effects of HcuAV In Vitro

#### 2.5.1. Neutralization against Neurotoxic Activity

**Figure 6:**
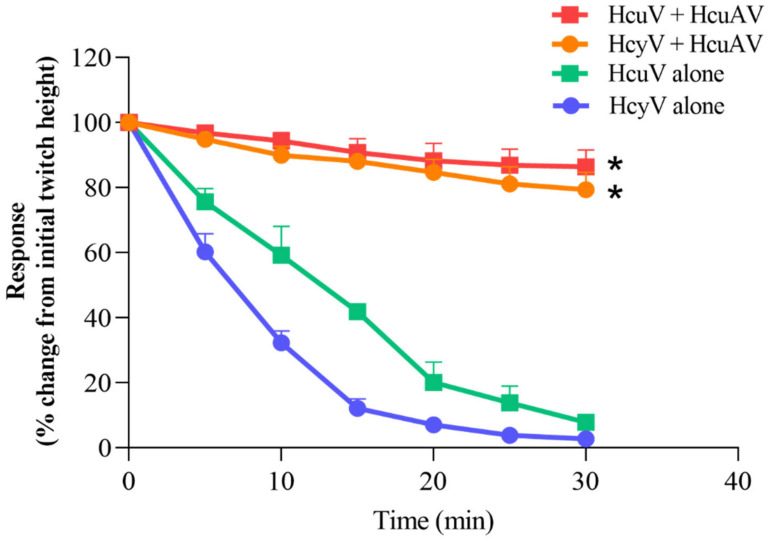
Neutralization of HcuAV against the neurotoxic effect of HcuV and HcyV. Briefly, the sciatic-nerve-gastrocnemius-muscle of a frog was dissected and mounted in tissue bath. Then, HcuAV and venoms diluted in Ringer’s solution were added at different groups. The venom concentration was 10 μg/mL (total volume: 10 mL), and the dose of antivenom (HcuAV) was 100 μL (1μL HcuAV/ μg venom). Twitches were evoked by stimulating the motor nerve, detected by a tension sensor, and recorded every 5 min in 30 min. The neuromuscular response was expressed as rate (%) of the initial twitch height. All treatments were performed in triplicate individually, and the data are presented as means ± SD (n = 3). (* *p* < 0.05 vs. venom alone group).

Neurotoxins are the predominant components in sea snake venoms, which can bind to muscle nicotinic receptors and inhibit acetylcholine from binding to the receptor, resulting in impaired neuromuscular transmission. Therefore, the neurotoxicity of sea snake venom mainly manifested as a neuromuscular blocking effect. To determine the neutralization effect of HcuAV, an assay on neuromuscular response was performed. Briefly, the sciatic-nerve-gastrocnemius-muscle of a frog was dissected and mounted in tissue bath. Then, HcuAV and venoms diluted in Ringer’s solution were added. Twitches were evoked by stimulating the motor nerve and detected by a tension sensor and recorded every 5 min for 30 min. The neuromuscular response was expressed as rate (%) of the initial twitch height. As shown in Figure 6, the twitch response of frog sciatic-nerve-gastrocnemius-muscle preparation was significantly weakened when treated with HcuV and HcyV, indicating that the two sea snake venoms had specific neuromuscular blocking effects. However, the twitch response of the preparation was only slightly weakened when treated with the mixtures of sea snake venom and HcuAV, indicating that HcuAV could effectively neutralize the neurotoxic activity of HcuV and HcyV.

#### 2.5.2. Neutralization against Venom Lethality

**Figure 7:**
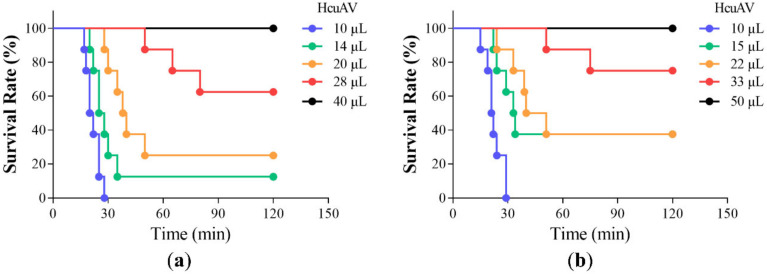
Survival curve of mice after the injections of mixtures. The mixtures contained various doses of HcuAV and a constant amount (4 × LD_50_) of HcuV (**a**) or HcyV (**b**). They were incubated at 37 °C for 45 min, and then the mixtures were injected intraperitoneally (i.p.) into mice. The number of deaths for each group was observed over a period of 24 h, and the survival curve of the mice was drawn. All treatments were performed in triplicate individually.

To determine the effective dose of HcuAV against HcuV and HcyV, different doses of HcuAV were incubated with a 4 × LD_50_ dose of HcuV and HcyV, and then the mixtures were injected intraperitoneally (i.p.) into mice. The number of deaths for each group was observed over a period of 24 h, and the survival curve of the mice was drawn. The ED_50_, defined as the amount of HcuAV against a 4 × LD_50_ dose of venoms that keeps half the mice alive, was determined using the Spearman Karber method. As shown in Figure 7a,b, the survival rate of mice significantly increased with the increase of doses of HcuAV. The ED_50_ of HcuAV against a 4 × LD_50_ dose of HcuV and HcyV were 24 μL and 22.3 μL, respectively, and the ER_50_ of HcuAV against HcuV and HcyV were 1.2 mg HcuV/1 mL HcuAV and 0.9 mg HcyV/1 mL HcuAV, respectively. These results provided a reference for the HcuAV dosage in future clinical application.

### 2.6. Time-Effect of HcuAV against Sea Snake Venoms In Vivo

**Figure 8:**
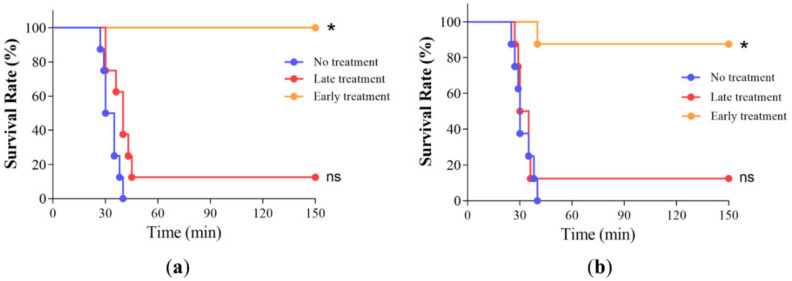
Survival curve of the envenomated mice with treatment of HcuAV at different time points. Briefly, sufficient HcuAV was injected intravenously (i.v.) into envenomated mice at different times (early or late period). The number of deaths for each group was observed over a period of 24 h, and the survival curve of the mice was drawn. All treatments were performed in triplicate individually. (**a**) HcuV; (**b**) HcyV. (* *p* < 0.05 vs. no treatment group).

To examine the effectiveness and dosing time of HcuAV in vivo, a mouse envenomated model was used. Briefly, sufficient HcuAV was injected intravenously (i.v.) into envenomated mice at 5 min (early treatment) or 20 min (late treatment) after envenomation to observe the treatment effects of HcuAV. The number of deaths for each group was observed over a period of 24 h, and the survival curve of the mice was drawn. As shown in Figure 8, for both HcuV and HcyV envenomation, the survival rate of the early treatment group was significantly higher than that of the late treatment group. In contrast, the survival rate of the late treatment group was not obviously different from that of the non-treatment group. These results indicated that an adequate dose of HcuAV should be injected as soon as possible after sea snake bites to effectively reduce the mortality rate.

### 2.7. Protective Effects of HcuAV on Organ Damage

**Figure 9:**
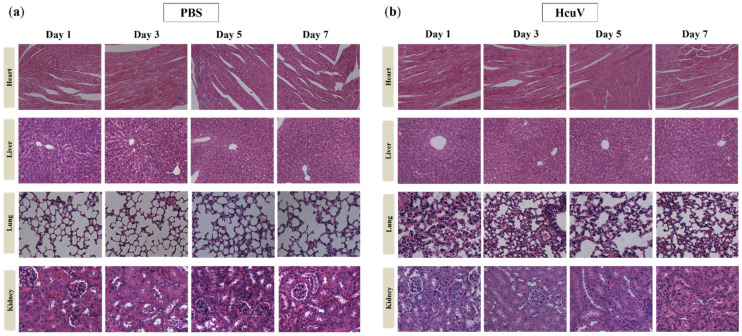
The heart, liver, lung, and kidney pathology of PBS-injected mice (**a**) and HcuV-envenomated mice (**b**). Briefly, non-lethal doses of HcuV (1/2 × LD_50_) and PBS were injected intravenously into mice, respectively. The mice were then sacrificed in 1, 3, 5, and 7 d, and the heart, liver, lung, and kidney were extracted for staining to observe the pathological changes.

First, to detect the organ damages caused by sea snake venoms, non-lethal doses of HcuV (1/2 × LD_50_) and PBS were injected intravenously (i.v.) into mice, respectively. The mice were then sacrificed in 1, 3, 5, and 7 d, and the heart, liver, lung, and kidney were extracted for staining to observe the pathological changes of the organs. As shown in Figure 9a,b, compared with the PBS injected group, there were no marked damages in heart and liver tissues of HcuV envenomated mice from d 1 to d 7 after envenomation. However, one day after envenomation, the lung tissue of envenomated mice manifested marked damage, and the damage had not recovered until d 7. In addition, three days after envenomation, the kidney tissue of mice exhibited obvious damage, and the damage gradually increased from d 3 to d 7. The above results demonstrated that the lung and kidney were the two main target organs damaged by sea snake venoms.

**Figure 10:**
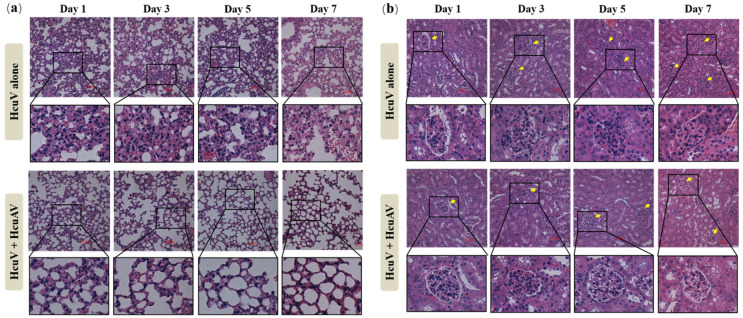
Protective effects of HcuAV on organ damage caused by sea snake venoms: (**a**) lung; (**b**) kidney, glomerulus (yellow arrow mark). Briefly, sufficient HcuAV was injected intravenously into HcuV-envenomated mice within a short period. The mice were then sacrificed in 1, 3, 5, and 7 d after treatment, and the lung and kidney were extracted for staining to observe the pathological changes.

To explore the protective effects of HcuAV on organ damage caused by a non-lethal dose of sea snake venom, sufficient HcuAV was injected intravenously (i.v.) into HcuV-envenomated mice within a short period. The mice were then sacrificed in 1, 3, 5, and 7 d after treatment, and the lung and kidney were extracted for staining to observe the pathological changes. As shown in Figure 10a, compared with the HcuV-envenomated group, the alveolus injury of mice in the HcuAV-rescue group significantly reduced, indicating that HcuAV could effectively protect lung tissue from damage by HcuV.

As shown in Figure 10b, 3 d after envenomation (without rescue), the kidney tissue of mice was obviously damaged, and it mainly manifested as an increase of the volume of glomerulus (yellow arrow mark), disappearance of renal capsules, and tubule lumens. Compared with the HcuV-envenomated group, the kidney injury of mice in the HcuAV-rescue group significantly reduced, indicating that HcuAV could effectively protect kidney tissue from damage by HcuV.

## 3. Discussion

In China, sea snakes are mainly distributed in the warm waters along the southeast coast [23], and sea snake bites often occurred when they were caught or when their habitats were disturbed [30]. Despite the low amount of expelling venom, the lethality of sea snake bites is very high due to the extreme toxicity of its venom [16]. In China, there is no commercially available sea snake antivenom, the most effective first aid for sea snake bites [31]. Therefore, it was necessary to perform the current study.

Sea snake venoms are a complex mixture of neurotoxins, enzymes, and small molecules [13,29,32]. The toxic effects of sea snake bites are caused by a combined action of the mixed components in the venom [20,33,34]. Therefore, to achieve better neutralizing effects, it is necessary to choose the whole sea snake venom as an antigen to prepare the antivenom. *Hydrophis curtus* and *Hydrophis cyanocinctus* are the two dominant species of sea snakes in Chinese seas [23]. The proteomic analysis in a previous study [23] has showed that HcuV and HcyV were similar in composition, except for a greater compositional diversity in HcuV than in HcyV. Therefore, in order to obtain a better neutralization effect, we chose the whole HcuV as the immunogen to prepare sea snake antivenom.

The concentration of formaldehyde for venom inactivation is a key factor affecting the immunogenicity of HcuV. We finally determined that HcuV inactivated with 0.6% formaldehyde (*v/v*) and emulsified in incomplete Freund’s adjuvant (IFA) exhibited the greatest immunogenicity and could obtain the highest titer after immunizing horses. The Fc fragment of intact IgG in plasma exhibits a complement activation effect, which can induce the formation of immune complexes, leading to allergic reactions [35,36]. Moreover, the protein fragments after enzyme digestion significantly affect the purity and titer of the final F(ab′)_2_ antibody. Therefore, the removal of intact IgG and digested fragments is important in the production and purification of antivenom. After a series of procedures, including pepsin digestion, ammonium sulfate precipitation, alum precipitation, ultrafiltration, and column chromatography, a high-purity HcuAV was obtained. In order to evaluate the neutralizing effects of HcuAV, we conducted a cross-immunization assay of HcuAV towards various snake venoms by ELISA and DID, and the results showed that HcuAV could effectively cross-neutralize venoms from the two dominant species of sea snakes in Chinese seas.

Due to the lack of sea snake antivenoms in China, BmAV was often used for treatment after sea snake bites. Our results confirmed that the neutralization ability of BmAV against HcuV and HcyV was weak, and the HcuAV prepared in the present study exhibited obvious advantages in neutralizing sea snake venoms. In addition, consistent with previous reports [23,37,38], the main components of HcuV and HcyV were neurotoxins (6–10 kDa) and PLA2s (13–16 kDa), and HcuAV could effectively bind the two groups of proteins to achieve the neutralization effect.

We further explored the effective dosage and timing of HcuAV for treatment after sea snake bites in mouse models, and the neutralizing potency of HcuAV against a 4 × LD_50_ dose of HcuV and HcyV, expressed as ED_50_, was determined. The results of in vitro neutralization experiments illustrated that 1 mL of HcuAV could effectively neutralize 1.2 mg of HcuV or 0.9 mg HcyV. The results of in vivo neutralization experiments confirmed that the injection of sufficient HcuAV as soon as possible after sea snake bites could significantly improve the survival rate of envenomated mice. At the same time, we also found that non-lethal doses of sea snake venom could induce irreversible damages to lung and kidney tissues, and these organ damages could be effectively prevented by HcuAV. However, the experimental envenomation in mice may not be reflective of human envenoming. More studies in large animal models are needed to explore the therapeutic effect and dosages of HcuAV for promoting its clinical application.

It should be noted that although antivenom is the most effective way to treat snakebites up to now, it also has various possible side effects, such as anaphylactic shock, pyrogen reaction and serum sickness. In addition, apart from the high preparation cost of investment and time [26,39,40], great distress to the employed animals, caused by frequent immunization and bleeding during antivenom production, cannot be ignored [41]. Therefore, with the continuous advancement of drug development technologies, such as antibody technology, phage display technology, and protein editing technology, the next generation of antivenom drugs should be developed rapidly [42,43,44,45,46].

## 4. Conclusions

In this study, we chose HcuV as the antigen to immunize horses and prepared a high-titer, high-purity sea snake antivenom after a series of procedures. HcuAV could effectively cross-neutralize lethal and neurotoxic activities of HcuV and HcyV. In addition, adequate administering of HcuAV as soon as possible could significantly reduce the mortality rate of sea snake bites and prevent damages to vital organs. These findings enhanced our understanding on the protective effects of sea snake antivenom and would provide important experimental evidence for the future clinical application of HcuAV.

## 5. Materials and Methods

### 5.1. Preparation of HcuV Antigen and Immunization of Horse

The preparation of HcuV and HcyV was the same as in our previous study [23]. Briefly, adult individuals of *Hydrophis curtus* and *Hydrophis cyanocinctus* were captured in the coastal waters of Haikou, Hainan Province (China) in October 2018. They were decapitated, and the venom glands were removed by dissection and then carefully blotted with blotting paper. The venom glands were then thoroughly pulverized with an electric grinder, and the venoms were extracted with distilled water. Insoluble tissue debris was removed by centrifugation, and the supernatant liquid was lyophilized. The lyophilized powders were named as HcuV and HcyV. The sea snake venoms used in this study were the same batch in our previous study, and the LD_50_ values of HcuV and HcyV were 0.34 μg/g and 0.24 μg/g, respectively [23].

The dry powder of HcuV was dissolved in Tris and centrifuged at 4000 rpm for 30 min to remove the precipitate. HcuV was inactivated using 0.6% formaldehyde solution (*v/v*) and then mixed with the incomplete Freund’s adjuvant at a final concentration of 5 mg/mL. The horses used for plasma production were purchased from pastures in the epidemic-free area of Gansu province, China. Healthy male horses aged 4–15 years, weighing 250–350 kg, were selected. The horses were strictly managed in accordance with the Regulations on Quarantine and Immunization of Horses for the Production of Immune Serum in the Chinese Pharmacopoeia. Each horse (numbered 1, 2, 3, 4, respectively) was immunized by multipoint subcutaneous injection with 1 mL (5 mg/mL) of HcuV mixture each time. After the first injection, intensified injections were given every 10 days for a total of five times.

### 5.2. Immune Titer Detection and Cytotoxicity Assay of the Immune Plasma

Blood samples (1 mL) were collected 10 d after each immunization into an anticoagulation tube containing 4% sodium citrate (1:16), and the plasma was obtained after centrifugation. Each well of 96-well plates had 100 μL of diluted HcuV (10 μg/mL in coating buffer) added and coated overnight at 4 °C. After pouring out the coated protein, each well had 200 μL of 2% bovine serum albumin added (BSA, dissolved in PBS with 0.05% Tween) at 37 °C for 1 h. The collected horse plasma was diluted at 1:20,000 with 2% BSA and was added to each well at 37 °C for 1 h. After fully washing, HRP-labeled anti-horse IgG diluted at 1:5000 was added and incubated at 37 °C for 1 h. Tetramethylbenzidine solution (TMB) was added to each well after washing, and absorbance at 450 nm was measured using a microplate reader (BioTek, Winooski, VT, USA).

Vero cells (African green monkey kidney cells) were seeded in a 96-well plate at 1 × 10^4^ cells/well and cultured overnight. The cells were treated with different dilutions of the horse plasma (at 1:5, 1:10, 1:20, 1:40) for 12 h. Then, 10 μL of CCK-8 reagents was added to each well for 2 h at 37 °C. Absorbance at 450 nm was measured using a microplate reader. Cell viability was calculated using the following formula:

Viability (%) = (OD_Treated_ − OD_Blank_/OD_Untreated_ − OD_Blank_) × 100

### 5.3. Purification of Antibody

Anti-venom immunoglobulins were prepared following several steps. First, 3000 mL of plasma was collected from each horse 10 d after the fifth immunization and then mixed with 2–4 times deionized water and the pH adjusted to 2.8–3.2. Second, pepsin (6–12 U/mL) was added to digest plasma at 30 °C for 60–90 min and mixed with 15% ammonium sulfate (*w/v*), and the pH was adjusted to 4.8–5.6. Third, the temperature was raised to 58 °C, and the supernatant (supernatant I) was filtered after stirring for 30 min. Then, the pH of the filtrate was adjusted to 7.0–7.4, followed by the addition of 20% ammonium sulfate (*w/v*), and then well stirred and allowed to stand still for 30–60 min. After filtering procedures, the precipitate (secondary precipitation) was collected. After dissolving the precipitate with 2–4 times deionized water, 80 mL/kg of 10% alum solution was added, and the pH of mixture was adjusted to 7.7–7.9. The supernatant (supernatant II) was stirred for 30 min and then filtered by plate and frame filtration. The filtrate was subjected to ultrafiltration to remove salt, so that the content of ammonium sulfate was less than 0.5 g/L. Then, a 10% volume of 0.2 M phosphate buffer was added to the ultrafiltrate, and the flow-through fluid was collected after DEAE column chromatography to obtain the refined IgG antibody. Finally, NaCl was added to the flow-through solution (8.5 g/L), and the pH was adjusted to 6.0–7.0. The antivenom stock solution was obtained after filtering and sterilizing through a 0.22 μm filter and stored at 2–8 °C in the dark.

### 5.4. Binding Assay of HcuAV towards Snake Venoms

#### 5.4.1. Double Immunodiffusion (DID) Assay

The binding ability of HcuAV to various snake venoms was determined by a DID assay, according to the specifications of Restrepo et al. [47,48]. One assay was performed with a series of diluted HcuAV (two-fold dilutions starting with 1:2) against HcuV and HcyV. The other assay was performed with undiluted HcuAV against HcuV, HcyV, and four types of terrestrial snake venoms, including *Naja atra* venom (NaV), *Bungarus multicinctus* venom (BmV), *Agkistrodon halys* venom (AhV), and *Deinagkistrodon acutus* venom (DaV).

#### 5.4.2. ELISA Assay

The binding ability of HcuAV and three types of terrestrial snake antivenoms towards various snake venoms were, respectively, determined by indirect ELISA assay. All tests were performed at the same place, and the samples were processed in triplicate.

Each well of 96-well plates had 100 μL of diluted venoms (10 μg/mL in coating buffer) added and coated overnight at 4 °C. After pouring out the coated protein, each well had 200 μL of 2% BSA (dissolved in PBST) added at 37 °C for 1 h. Then, different dilutions of HcuAV and three terrestrial antivenoms were added to each well at 37 °C for 1 h. After fully washing, an HRP-labeled antibody was added and incubated at 37 °C for 1 h. TMB was added to each well after washing, and an absorbance at 450 nm was measured using a microplate reader.

#### 5.4.3. Western Blot Assay

HcuV and HcyV were dissolved in PBS at a concentration of 1 mg/mL. Equal amounts of venom sample were loaded on a 12% SDS-PAGE gel and transferred to a polyvinylidene difluoride membrane, which was then blocked with 5% non-fat dry milk in TBST (3 g Tris-base, 8 g NaCl, 0.2 g KCl, 0.05% Tween-20, diluted with water to 1000 mL, pH 7.4) for 2 h at room temperature. The membranes were then incubated overnight at 4 °C with HcuAV and four types of terrestrial snake antivenoms at a dilution of 1:1000, respectively. The electrochemiluminescence method was used with a secondary antibody (HRP-conjugated anti-horse IgG) at a dilution of 1:5000 for 2 h at room temperature. Afterward, the membranes were exposed using a chemiluminescent detection system (Syngene G: Box, Cambridge, UK).

### 5.5. Neutralizing Effect of HcuAV against HcuV and HcyV

#### 5.5.1. Neutralization of Venom Neurotoxicity

Sea snake venoms mainly manifest a neuromuscular blocking effect. To determine the neutralization effect of HcuAV, we detected the neuromuscular response in different groups, using a previously described assay with minor modifications [28,49]. Briefly, the sciatic-nerve-gastrocnemius-muscle of a frog was dissected and mounted in tissue bath. HcuAV was diluted in Ringer’s solution (NaCl, 118.4; NaHCO_3_, 25; glucose, 11; KCl, 4.7; MgSO_4_, 1.2; KH_2_PO_4_, 1.2 and CaCl_2_, 2.5, mM), and then the venoms were added. The venom concentration was 10 μg/mL (total volume: 10 mL), and the dose of HcuAV was 100 μL (1μL HcuAV/1μg venom). Tissues were then bathed in Ringer’s solution, containing different mixtures (HcuV alone, HcyV alone, HcuV and HcuAV, HcyV and HcuAV). Twitches were evoked by stimulating the motor nerve at a voltage greater than that required to evoke a maximal twitch. The twitches were detected by a tension sensor and recorded every 5 min for 30 min. The neuromuscular response was expressed as rate (%) of the initial twitch height.

#### 5.5.2. Median Effective Dose

Neutralizing potency of HcuAV was expressed as ED_50_ (the volume of antivenom in μL that achieves a 50% survival rate) and ER_50_ (the ratio of the venom amount to ED_50_ when there was a 50% survival rate) [50,51]. To determine the ED_50_ of HcuAV against HcuV and HcyV, mixtures containing various doses of HcuAV and a 4 × LD_50_ dose of venoms were pre-incubated at 37 °C for 45 min, and the mixtures were intraperitoneally (i.p.) injected into four groups of mice (ICR, 18–20 g, 8 mice/ group). The number of deaths for each group was observed over a period of 24 h, and the survival curve of the mice was drawn. The ED_50_, defined as the amount of HcuAV against a 4 × LD_50_ dose of venoms that kept half the mice alive, was determined using the Spearman Karber method.

#### 5.5.3. Time-Effect of HcuAV against Sea Snake Venoms In Vivo

**Figure 11:**
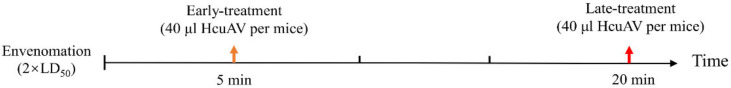
The time axis of envenomation and treatment.

To determine the time-effect of HcuAV on the mice envenomated with HcuV or HcyV (2 × LD_50_), sufficient HcuAV (40 μL per mice) was injected intravenously (i.v.) into envenomated mice at different times (early and late period) (3 groups of ICR mice, 18–20 g, 8 mice/ group). The time points of 5 min (early treatment) and 20 min (late treatment) after envenomation to observe the treatment effects of HcuAV were based on our pre-experiments. The time-axis of envenomation and treatment was shown in Figure 11. The number of deaths in each group was recorded over a period of 24 h, and the survival curve of the mice was drawn.

#### 5.5.4. Protective Effects of HcuAV on Organ Damage

To detect the organ damage caused by sea snake venoms, a non-lethal dose of HcuV (1/2 × LD_50_) and PBS were injected intravenously (i.v.) into mice. The mice in different groups were then sacrificed in 1, 3, 5, and 7 d, and the heart, liver, lung, and kidney were extracted for staining to observe the pathological changes of the organs.

To explore the protective effects of HcuAV on the mice envenomated with a sublethal dose of HcuV (1/2 × LD_50_), sufficient HcuAV was intravenously (i.v.) injected into 1/2 × LD_50_ of HcuV envenomated mice within 10 min. The mice were sacrificed by cervical dislocation in 1, 3, 5, and 7 d after treatment, respectively, and the lung and kidney tissues were extracted for hematoxylin-eosin staining to observe the pathological changes of the organs.

### 5.6. Statistical Analysis

The experimental data were analyzed using GraphPad Prism 8 statistical software. The comparison of the two groups of means was performed by *t* test, and the differences between three groups and more were performed using a one-way ANOVA test. A value of *p* < 0.05 was considered statistically significant. Data were presented as the mean ± SD, and the experiments were performed in triplicates.

### 5.7. Ethical Identification

All experimental animals in this study were kept in a pathogen-free environment and fed at lib. The procedures for care and use of animals were approved by the Committee on Ethics of Medicine, Navy Medical University, PLA in 5 March 2019, and the project identification code was 81974496.

## Figures and Tables

**Table 1:** Immune titer of the plasma obtained after each immunization of the horses.

Horse	Plasma 1 (10 d)	Plasma 2 (20 d)	Plasma 3 (30 d)	Plasma 4 (40 d)	Plasma 5 (50 d)
1	0.052	0.428	0.781	1.233	1.712
2	0.050	0.321	0.602	1.031	1.342
3	0.045	0.407	0.711	1.111	1.687
4	0.046	0.381	0.627	1.103	1.548

Each horse was immunized by a multipoint subcutaneous injection with 1 mL (5 mg/mL) of HcuV mixture each time. After the first injection, intensified injections were given every 10 days for a total of 5 times. The plasma was obtained 10 d after each immunization, and the immune titer was detected.

**Table 2:** Proportion of peak area of each sample of HcuAV during purification (%).

No.	Sample	IgG, Multimer (%)	F(ab’)_2_ (%)	Fragments (%) (Fc, Fab’, Albumin)
1	Enzyme digestion solution	17.8	19.8	62.4
2	Supernatant I after primary precipitation	2.6	42.8	54.6
3	Precipitation resuspension after secondary precipitation	3.5	57.7	38.8
4	Supernatant II after alum adsorption	0.02	76.3	23.6
5	Ultrafiltrate	2.9	84.5	12.6
6	Stock solution	1.2	91.6	7.2

## Data Availability

Not applicable.
